# Blind testing of shoreline evolution models

**DOI:** 10.1038/s41598-020-59018-y

**Published:** 2020-02-07

**Authors:** Jennifer Montaño, Giovanni Coco, Jose A. A. Antolínez, Tomas Beuzen, Karin R. Bryan, Laura Cagigal, Bruno Castelle, Mark A. Davidson, Evan B. Goldstein, Raimundo Ibaceta, Déborah Idier, Bonnie C. Ludka, Sina Masoud-Ansari, Fernando J. Méndez, A. Brad Murray, Nathaniel G. Plant, Katherine M. Ratliff, Arthur Robinet, Ana Rueda, Nadia Sénéchal, Joshua A. Simmons, Kristen D. Splinter, Scott Stephens, Ian Townend, Sean Vitousek, Kilian Vos

**Affiliations:** 1 School of Environment, Faculty of Science, University of Auckland, Auckland, 1010 New Zealand; 2 Departamento de Ciencias y Tecnicas del Agua y del Medio Ambiente, Universidad de Cantabria, Santander, Spain; 3 Water Research Laboratory, School of Civil and Environmental Engineering, UNSW, Sydney, 2052 Australia; 4 School of Science, University of Waikato, Private Bag 3105, Hamilton, New Zealand; 5 UMR EPOC, University of Bordeaux/CNRS, Bordeaux, France; 6 Coastal Processes Research Group, School of Biological and Marine Sciences, Plymouth University, Drake Circus, PL4 8AA Plymouth, UK; 7 Department of Geography, Environment, and Sustainability, University of North Carolina, Greensboro, NC 27412 USA; 8 BRGM, 3 avenue Claude Guillemin, 45060 Orléans cédex, France; 9 Scripps Institution of Oceanography, University of California, San Diego, United States; 10 Division of Earth and Ocean Sciences, Nicholas School of the Environment, Center for Nonlinear and Complex Systems, Duke University, Durham, NC USA; 11 U.S. Geological Survey St. Petersburg Coastal and Marine Science Center, 600 4th Street South, St. Petersburg, FL USA; 12 National Institute of Water and Atmospheric Research, Hamilton, New Zealand; 13 University of Southampton, Southampton, SO17 1BJ UK; 14 Pacific Coastal and Marine Science Center, U.S. Geological Survey, Santa Cruz, CA USA; 15 Department of Civil and Materials Engineering, University of Illinois, Chicago, IL USA

**Keywords:** Natural hazards, Physical oceanography

## Abstract

Beaches around the world continuously adjust to daily and seasonal changes in wave and tide conditions, which are themselves changing over longer time-scales. Different approaches to predict multi-year shoreline evolution have been implemented; however, robust and reliable predictions of shoreline evolution are still problematic even in short-term scenarios (shorter than decadal). Here we show results of a modelling competition, where 19 numerical models (a mix of established shoreline models and machine learning techniques) were tested using data collected for Tairua beach, New Zealand with 18 years of daily averaged alongshore shoreline position and beach rotation (orientation) data obtained from a camera system. In general, traditional shoreline models and machine learning techniques were able to reproduce shoreline changes during the calibration period (1999–2014) for normal conditions but some of the model struggled to predict extreme and fast oscillations. During the forecast period (unseen data, 2014–2017), both approaches showed a decrease in models’ capability to predict the shoreline position. This was more evident for some of the machine learning algorithms. A model ensemble performed better than individual models and enables assessment of uncertainties in model architecture. Research-coordinated approaches (e.g., modelling competitions) can fuel advances in predictive capabilities and provide a forum for the discussion about the advantages/disadvantages of available models.

## Introduction

Quantitative prediction of beach erosion and recovery is essential to planning resilient coastal communities with robust strategies to adapt to erosion hazards. Over the last decades, research efforts to understand and predict shoreline evolution have intensified as coastal erosion is likely to be exacerbated by climatic changes^1–5^. The social and economic burden of changes in shoreline position are vast, which has inspired development of a growing variety of models based on different approaches and techniques; yet current models can fail (e.g. predicting erosion in accreting conditions). The challenge for shoreline models is, therefore, to provide reliable, robust and realistic predictions of change, with a reasonable computational cost, applicability to a broad variety of systems, and some quantifiable assessment of the uncertainties.

Shoreline evolution occurs over temporal scales ranging from seconds (e.g., individual waves) to hours (e.g., storms), months (e.g., seasonal wave energy modulation) and decades (e.g., wave climate). Shoreline changes occurring over much larger timescales (decadal to centennial) can be the result of other factors like longshore sediment transport gradients, changes in sediment supply, tectonic processes, anthropogenic interventions, and sea level rise (SLR)^6–8^. Cross-shore sediment transport is generally considered to be the main control of shoreline evolution at seasonal and inter-annual time-scales^9,10^ whilst longshore processes (specifically on open coastlines) become more relevant over much longer timescales (decades-centuries)^7,11^.

**Figure 1:**
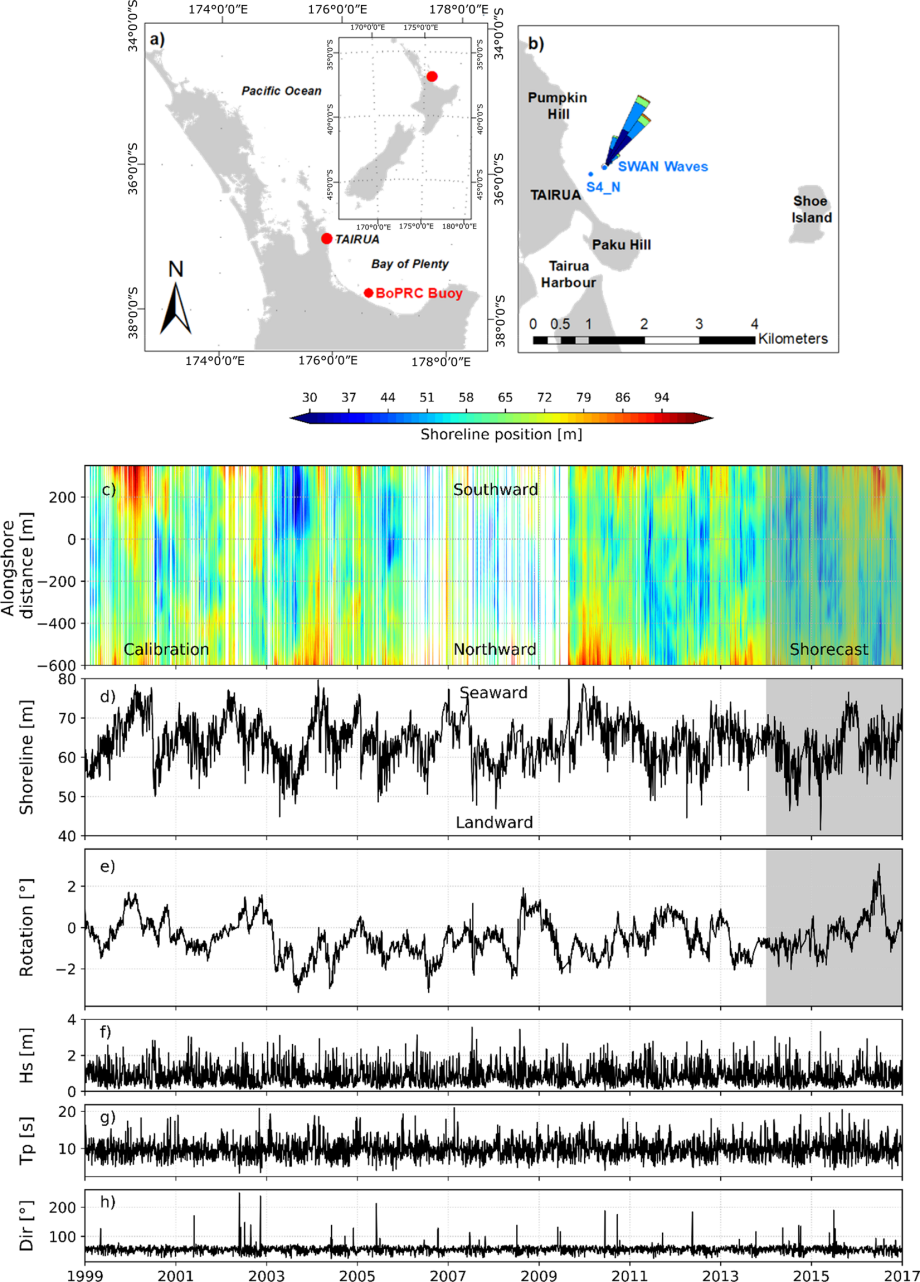
Study site and input conditions. (**a**) Location of Tairua, New Zealand North Island (**b**) Detail of Tairua Beach. Pressure sensor (S4_N) location used for SWAN model validation (**c**) Alongshore shoreline position at Tairua beach. Red represents shoreline advance and blue shoreline retreat over time. (**d**) Daily alongshore-averaged position (**e**) Shoreline rotation (orientation) with positive values representing southward accretion (anti-clockwise rotation) and negative values representing northward accretion (clockwise rotation). (**f**) Significant wave height g) Peak period h) Wave direction. Grey shading show the data that was hidden from modelers (*Shorecast* period, 2014–2017).

To test and improve the ability of models to predict shoreline changes, we carried out a workshop/competition on shoreline evolution modelling, “*Shoreshop”*, with participants from 15 institutions worldwide. Modelers were asked to simulate shoreline evolution obtained using a camera system at Tairua beach (New Zealand, Fig. 1a,b) and submit the results of the simulations without prior knowledge of how the shoreline actually evolved for the last 3 years (2014–2017) of the total study period (1999–2017). Data in the grey shading (Fig. 1c–e) were not shown to the modelers to ensure that they were not tempted to adjust parameters of their model framework after exposure to the results.

This article summarizes the main outputs from a study where well-known models that have been broadly used in diverse study sites worldwide^12–18^ combined with new approaches were compared and evaluated objectively and with no possibility of parameter tuning during the last three years of the study. As all the models are tested with the same input dataset, there is no bias associated with data sources (regardless of any inherent uncertainties in the data that was used) allowing us to objectively assess the predictive capability of the models. As shown in other disciplines^19^, modelling competitions are a powerful tool to promote advances since they favour research-coordinated approaches, and, importantly, encourage the community to share datasets to assess and compare models while ensuring reproducibility which allows for objective assessment.

## Data and Models

In this study we concentrate on models that make use a set of cross-shore profiles or shorelines captured from aerial or oblique photography to relate changes in the shoreline position to the prevailing forcing conditions. Although 3D digital terrain models are now becoming available, there are few long-term (multi-year) data sets of this type. Figure 1 shows the entire dataset used in the study (1999–2017). Shoreline position as a function of alongshore location and time is shown in Fig. 1c. Changes in the shoreline orientation, here referred to as shoreline rotation, evaluated as the slope of the trend-line fitted to the shoreline position before alongshore-averaging, are often observed (Fig. 1c,e). The average alongshore position (Fig. 1d) shows seasonality with progradation and retreat events generally occurring in summer and winter. The grey shading in Fig. 1c–e highlights the period hidden from modelers (2014–2017). Three hourly wave characteristics (wave height, peak period, and direction), obtained from a wave hindcast using the SWAN model forced with Wavewatch III model, are shown in Fig. 1f–h. More information about the study site characteristics and input data used during the study can be found in the Methods section.

We focus on daily shoreline predictions using a variety of modelling approaches (ranging from established shoreline models to Machine Learning algorithms), which, in the context of the 3 years of testing data (2014–2017), we hereafter referred to as the “*Shorecast*”. A total of 19 models were used. Twelve models (indicated with HM) were built following various formulations of the well-established equilibrium concept, where the beach rate of change is governed by the difference between present and equilibrium conditions^12,13^. Some of these models were also used to predict shoreline rotation (indicated with R). Seven models were built using Machine Learning techniques (indicated with ML). Table 1 summarises the models used during the study. More information about the models can be found in the Methods section and the supporting information.

**Table 1:** Models used during the “Shoreshop”.

	Model name/ Technique	Modeller
***Hybrid Models (HM)***		
HM1	ShoreFor	Kristen Splinter
HM2-R1	ShoreFor-LX	Mark Davidson
HM3	Y09-HF	Jennifer Montaño
HM4	ShoreFor + uKF	Rai Ibaceta
HM5	Y09	Bonnie Ludka
HM6, HM7	[-]	Ian Townend
HM8, R2	LX-Shore	Arthur Robinet, Bruno Castelle, Deborah Idier
HM9, R3	CosMos-Coast	Sean Vitousek
HM10, R4	COCOONED	Jose A. A. Antolinez
R5, R6	[-]	Karin Bryan
***Machine Learning (ML) Models***		
kNN	k- Nearest Neighbor	Evan Goldstein
ANN-EI1, 2	Autoregressive NN with exogenous inputs	Giovanni Coco
NeuFor	Artificial NN	Josh Simmons
LSTM	Long-Short Term Memory	Sina Masoud Anasari
RF	Random Forest	Tom Beuzen
BNN	Bayesian N	Nathaniel Plant

## Results

### Calibration period

**Figure 2:**
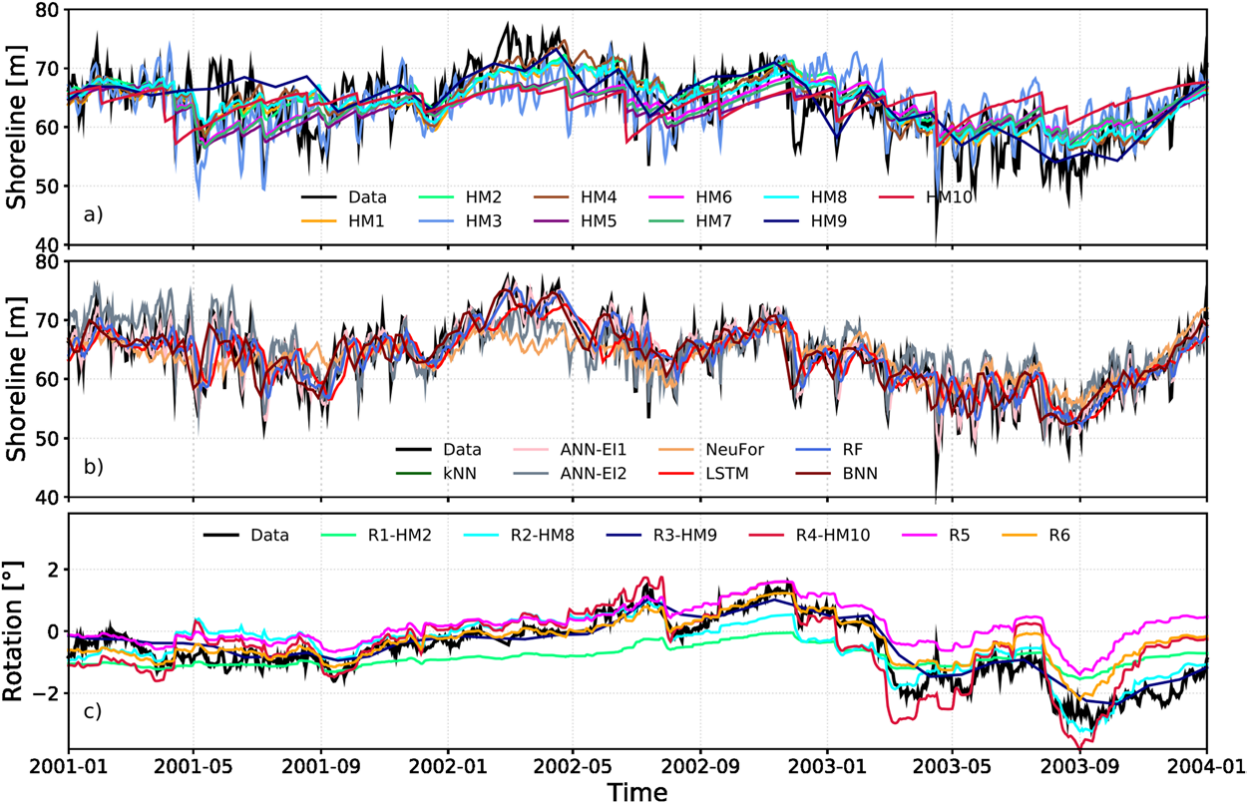
Three years of the entire calibration period (1999–2014). Examples of model outputs (see legend) compared to three years (2001–2004) of calibration data (black): (**a**) Hybrid models; (**b**) Machine Learning models; (**c**) Shoreline rotation models. See Methods section and Supporting Information for model details.

**Figure 3:**
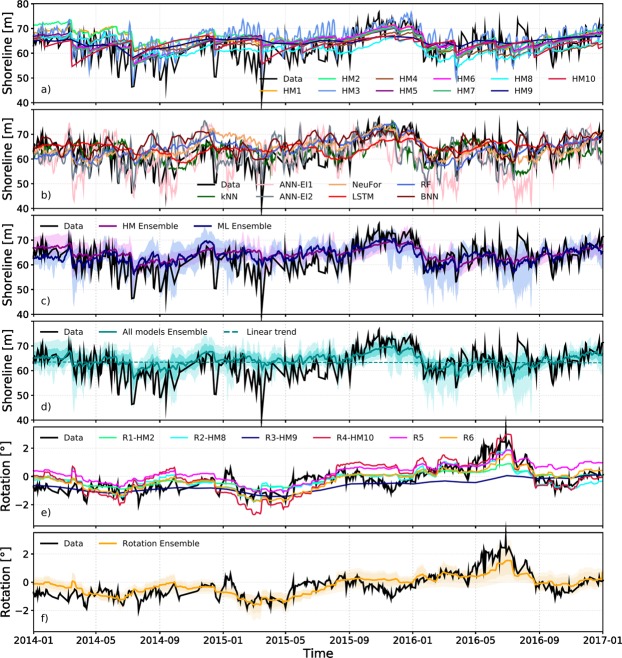
*Shorecast* predictions (2014–2017, blind test). Model outputs (see legends) compared to observations (black) (**a**) Hybrid models (**b**) Machine Learning models (**c**) HM and ML ensemble (**d**) Multi-model ensemble (**e**) Rotation models (**f**) Hybrid models ensemble for beach rotation. Dark shadows in the ensembles figures represent one standard deviation of the models prediction. Light shadows represent maxima/minima envelope of the models predictions. See Methods section and Supporting Information for model details.

**Figure 4:**
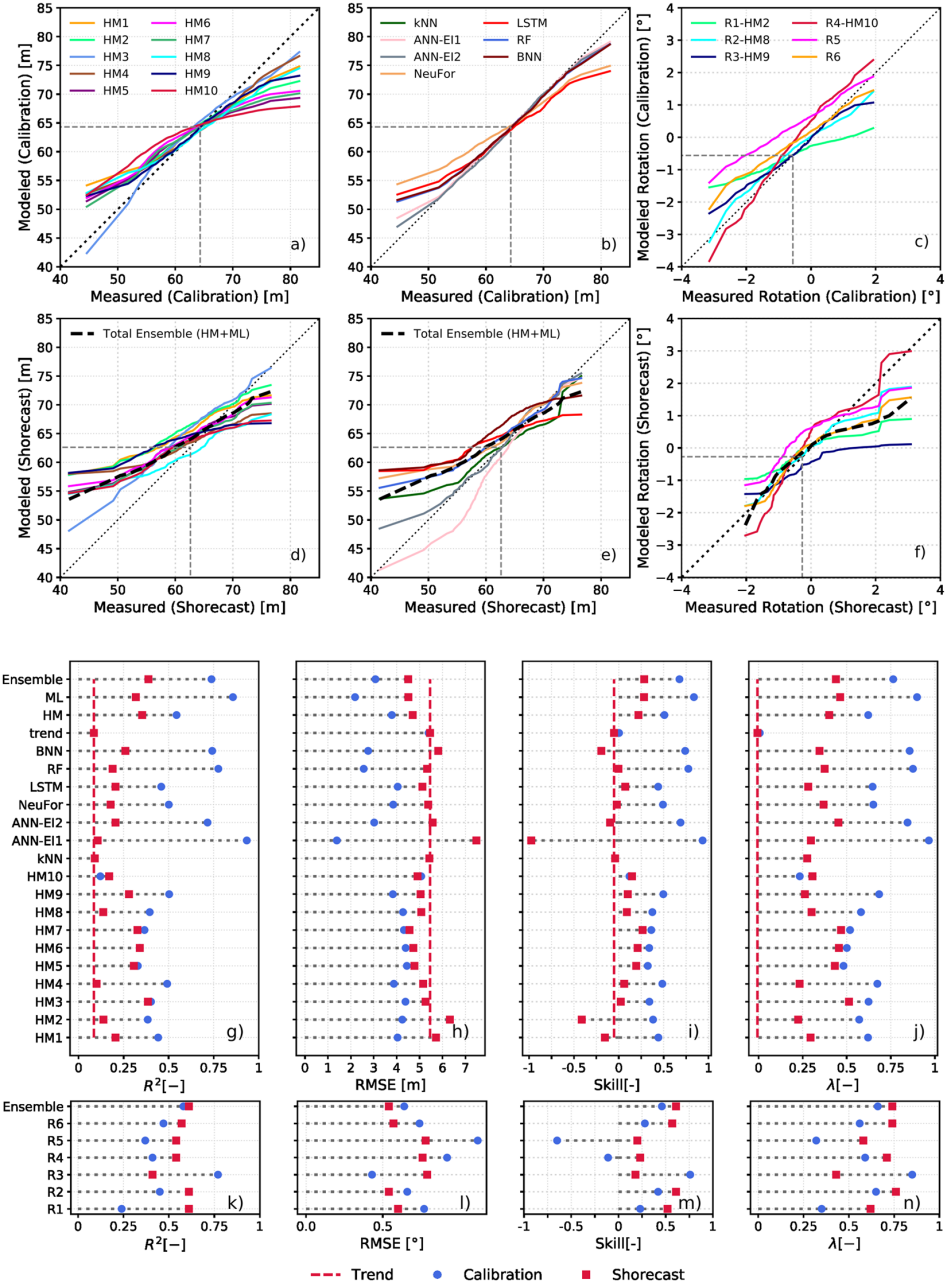
Models performance. Quantile-quantile plots of model behavior. Top 3 panels: Calibration period; middle 3 panels: *Shorecast*. Model prediction vs measured shoreline position for (**a**) and (**d**) HM; (**b**) and (**e**) ML; (**c**) and (**f**) model prediction vs measured shoreline rotation. Dashed grey line represent the average shoreline position during the calibration and the *Shorecast* period, respectively. R^2^, RMSE, skill and *λ* for shoreline prediction for; (**g**–**j**) averaged shoreline position; (**k**–**n**) shoreline rotation. See supporting material for more information about the metrics and individual models.

Fifteen years (1999–2014) were used for model calibration (Fig. 2), while the last three years (2014–2017) were used for the blind prediction, *Shorecast* (Fig. 3), in which modelers did not have access to the shoreline data. Both HM and ML approaches were able to reproduce seasonal cross-shore (alongshore averaged) shoreline behaviour during the calibration period. Figure 2 shows the models’ performance for three years (2001–2004) of the calibration period (1999–2014). Regardless of the modelling approach, oscillations of shoreline position with periods larger than 3 months were well captured. Some of the HM and almost all ML models were able to reproduce accretion periods (e.g. beginning of 2002, Fig. 2a,b). In general, HM predictions were smoother than ML predictions which reproduced faster oscillations (shorter than seasonal) and more extreme events in the shoreline position (Figs. 2b and 4b). For instance, almost all HM underestimate the erosion that occurred on August 2003 (Fig. 2a) except for the models that use data assimilation (Kalman filters, HM4, HM9). There were no major differences among HM that sought to define an equilibrium condition using: wave history (HM1, HM2, HM4 and HM8) or shoreline position (HM3, HM5, HM6, HM7, HM9 and HM10). HM models improved when Kalman filters were used (HM4 and HM9). Despite being diverse in approach and architecture, all ML models displayed very high performance during the calibration period compared with the *Shorecast* period.

Some of the HM (HM2-R1, HM8-R2, HM9-R3 and HM10-R4) and others specifically developed for shoreline rotation (R5, R6) were also used to predict beach rotation (evaluated as the slope of the trend-line fitted to the shoreline before alongshore-averaging), but no ML model was tested. Some of the rotation models essentially simulate multiple 1D cross-shore models which then require multiple calibration of cross-shore profiles along the embayment (see the Supporting Information). Almost all the models followed the rotation pattern (clockwise or anticlockwise) even during extreme rotation events. An exception is R1, which displayed a smooth behaviour (Fig. 2c and qq-plot Fig. 4c). Conversely, some models consistently over-estimate rotation (e.g., R4).

#### Shorecast (Three years of blind prediction)

During the *Shorecast* (last three years of unseen data, grey shading Fig. 1c–e), HM collectively displayed similar behaviour (Figs. 3a and 4d) and this was distinct from the ML models (Figs. 3b and 4e). Both types of models were able to predict the seasonal changes. Underestimation of extreme erosion events by HM (models above dotted black line, Fig. 4d) and inability of reproducing faster scale oscillations (order of 30 days) were more evident during the *Shorecast* (for example, see the beginning of 2015, Fig. 3a). Some of the ML models captured extreme accretion-erosion events and faster scale oscillations not reproduced by HM. However, the models that better captured some localised large shoreline changes were also the ones that for other events produced the largest errors (Figs. 3b and 4e). Results from ML models changed more from the calibration to the testing phase compared to HM results (Figs. 3b and 4b,e), except for the HM that used Kalman filters which had a similar behaviour to the ML models. During the *Shorecast* period, the mean of the averaged alongshore shoreline position was slightly different to the mean for the calibration period (dashed grey lines in Fig. 4a–f). Models tend to follow the calibration period mean shoreline position during the *Shorecast*, suggesting they are heavily dependent on the training dataset, which may indicate that they cannot predict/follow the long-term trend underlying the short term changes.

All models capture the general rotation patterns (shoreline orientation clockwise/anticlockwise) during the *Shorecast*, showing a better performance in terms of metrics than during the calibration period, except for the models that used data assimilation (Figs. 3d and 4). This may suggest that models have less skill for extreme rotation events since the *Shorecast* period showed fewer and smaller beach rotation events compared with the calibration period. At times, models were able to predict shoreline rotation but underestimated/overestimated the shoreline position. Conversely, the shoreline rotation was poorly predicted at times when the erosion and accretion events were reasonably predicted. In fact shoreline advance or retreat is computed using the average alongshore shoreline position, while beach rotation (change in the shoreline orientation) considers all the alongshore transects in the trend-line fit.

### Model ensembles

Uncertainties due to the model limitations have been addressed through multi-model ensembles^20^, such as, the global climate models^21,22^. An ensemble of the HM and ML models was created as a mean estimate of each type of model (HM and ML models, separately) to compare them (Fig. 3c).

ML models displayed comparable skill to HM, suggesting they might be useful in describing multi-year variability at shorelines not well simulated by HM. In general, HM predictions do not capture the extremes events in shoreline position that occur over short time-scales (~monthly) exhibited by the Tairua beach data. In contrast, ML reproduced these fast oscillations and the more extreme events in the shoreline position (Figs. 2b and 4b). Overall, it appears that these two classes of model tend to focus on different timescales, even though time-scale is not explicitly controlled in many of the models used. Therefore, ML models and HM may play complementary role in estimating cross-shore shoreline position, due to their different approaches (inductive versus deductive). A multi-model ensemble is generated as a mean estimate of all the models (Fig. 3d and dashed black line in Fig. 4d,e). The total multi-model ensemble *Shorecast* often overlaps the shoreline data, showing capacity to predict seasonality and some extreme events, for instance, accretion (end of 2015) and erosion (beginning of 2016). When all models reproduce the measured shoreline position correctly, the average of the models converges (low standard deviation), while when some of the models diverge from the measured shoreline, the ensemble cancels out the possibility of a large error. In general, ensembles (HM, ML and all models ensemble) showed better performance than many individual models (Figs. 3c,d and 4 and Supporting Information-Table 1). Even though an ensemble approach may increase model complexity and might smooth the predictions, Fig. 4d,e show that the total model ensemble captured extreme events (erosion/accretion) better than almost all HM and some of the ML models. Therefore, the ensemble approach improves the reliability of the predictions, and in effect reduces model-related uncertainty (Fig. 3d,f).

## Discussion

### Assessment of models performance

We calculated different metrics to assess predictive model performance (Fig. 4g–n and Supporting Information). We included a linear trend as a predictive model and, even though the linear trend does not follow the shoreline oscillations, metrics like R^2^, RMSE or Skill were better than for some of the models (Fig. 4 and Supporting Information Table 1).

The best metric for assessing model performance remains unclear and different model performance metrics favoured different models, highlighting the importance of considering multiple metrics and different approaches for a robust model evaluation. Model performance was also assessed in terms of quantile-quantile (predictions vs measurements) which provides information about extremes events, the direction of shoreline change (erosion/accretion) and mean behaviour (Fig. 4a–f). In addition, we acknowledge that the usefulness of a model should not be expressed only in terms of metrics but also reproducibility and understanding that leads to scientific advancements.

In general, models showed a lower performance during the *Shorecast* period than during the calibration period, and lower performance compared to previous studies in different sites where the models were first presented and tested against a data set^12–18^. The exact reason is difficult to determine but it is evident that, despite data uncertainties, true predictions of unseen shoreline data remains a difficult task.

### Uncertainty

Uncertainties are a key component of any modelling study. During the *Shorecast*, uncertainty arises from both the shoreline position and wave characteristics. Wave characteristics have been obtained through a numerical model and so contain potential sources of error. This could affect models differently since some models only use wave height and others include also the wave period which in general is more difficult to reproduce. Despite the wave period being poorly reproduced when compared to reproducibility of the wave height (See Methods), this did not seem to affect models’ performance during the calibration. Due to the stochastic nature of waves, probabilistic approaches using synthetic hydrodynamic forcing may give more realistic shoreline predictions that account for uncertainties, especially for long-term projections^23,24^. The shoreline positions we provided are likely to contain detection inaccuracies, although most of the errors occur over a scale shorter than a week^25^. Large shoreline changes, order of 30 days, were observed and these changes are only marginally affected by the faster-scale inaccuracies^25^. This is relevant since a number of models managed to reproduce instances of rapid and large shoreline retreat, but others completely missed the fast, order of 30 days, shoreline changes.

A different kind of uncertainly involves model structure and parameterizations. Such uncertainties in shoreline models arise because we use simplified models that may ignore some of the physical processes; for example, HM used during the *Shorecast* lack processes including, for example, overwash, beach-dune and/or beach-cliff interactions, influence of bars, human interventions which may play an important role in shoreline evolution. Also, we use a variety of ML techniques to find hidden processes and relations among drivers and response since, *a priori*, it is uncertain which ML model works best for the dataset provided. There is also an uncertainty related to parameters. While some models used only a minimal number of parameters (e.g., equilibrium models), others included many more (e.g., ML models) which brings up questions about model generality. Our test indicates that, even when model structure is similar, results may differ because models are highly dependent on a range of parameters that the scientist chooses.

### Long-term predictions

Uncertainty in drivers (e.g., waves and SLR) and therefore shoreline response increases as longer time horizons are explored. During *“Shoreshop”*, we also attempted to predict Tairua shoreline evolution until 2100. Results are not presented here because uncertainties in the future wave climate were deemed too large and the model outputs diverged drastically. However, the exercise was educational in two ways. First, it highlighted that shoreline evolution models require calibration and often fail when implemented outside of the regime of calibration. Secondly, the processes causing long-term shoreline change can differ from those that produce seasonal to multi-annual oscillations in shoreline position or plan-view shape (as addressed in the calibration and *Shorecast* periods). For an alongshore-restricted beach like Tairua, cross-shore processes related to SLR are likely to be the main cause of cumulative shoreline change. However, many of the models presented do not include the effects of SLR. We have thus far discussed creating ensembles for shoreline prediction, but ensembles are also needed for the drivers such as, SLR, wave conditions, storm surge where different scenarios are considered^8,23,24^.

Comparisons between multiple models on additional datasets (e.g. longshore vs cross-shore transport or seasonal vs storm dominance) are recommended to address issues in long term predictions that include (a) downscaling an appropriate future wave climate using a probabilistic approach that allows uncertainties to be accounted for, (b) assessing morphodynamic implications of different scenarios of SLR, and (c) dealing with the effect of out-of-calibration parameters. This will help better differentiate model performance, including transportability to different wave climates and morphodynamic settings. We foresee an increase of this type of modelling competition as a way to accelerate knowledge exchange and dissemination, as well as fostering community interaction.

## Methods

### Study site

Tairua Beach is located in the Coromandel peninsula, on the east coast of the North Island of New Zealand (Fig. 1a,b). Tairua is a pocket beach of 1.2 km long, with medium to coarse sand that exhibits intermediate beach states^25–27^. The lower shoreface slope is approximately 0.02, whereas the upper beach slope is steep ≈ 0.2^28^. The beach is located in a microtidal environment with a tidal range varying between 1.2 and 2 m. Eighteen years of daily shoreline evolution (1999–2017) were obtained using a camera system located on a hill (elevation about 60 m) at the north end of the beach. During daylight, six hundred images were averaged over a period of 15 min every hour. The time-averaged images were then georectified and used to extract the shoreline position. To limit the influence of the tides, daily shoreline images with tidal level between 0.45 and 0.55 m were selected. Errors in the shoreline detection due to the footprint of the georectified images, standard deviation of the water levels, the influence of the tides, the uncertainty in wave setup, and other noise have been shown to affect the daily timescale but not the shoreline signal over weekly (and longer) timescales^25^. The wave characteristics (at 10 m water depth) were obtained using a hydrodynamic model (SWAN), validated with *in situ* measurements in 8 m water depth (indicated as S4_N in Fig. 1b). The comparison was good in terms of wave height (*R*^2^ = 0.80 and *RMSE* = 0.31 *m*) but the wave period was poorly reproduced (*R*^2^ = 0.22 and *RMSE* = 0.29 *s*). The instrument used did not record wave direction so that a direct comparison could not be made.

### Model classification

We have classified shoreline models as: process-based models (PBM), hybrid models (HM), and data-driven models (DDM). The PBM are simulation models or physics-based models, which include as many processes as practicable, and usually couple hydrodynamics, waves, sediment transport and morphology through mass and momentum conservation laws. In general, these models attempt to describe the faster (storm event) and smaller scale (<km) processes and there is no conclusive evidence they can be successfully applied over large spatio-temporal scales. Also, in many cases, these models require input data that were not available for the present study.

We use the term “hybrid” to characterize shoreline models based on general principles (e.g., that a system is drawn towards an equilibrium configuration) that do not use detailed conservation of mass and momentum equations, and rely heavily on a data-driven approach to find the free parameters of the model. HM often base the prediction of the cross-shore position on the equilibrium concept^29^, where the beach rate of change is governed by the difference between present and equilibrium conditions. Equilibrium conditions have been defined in terms of shoreline position^10,12^ or wave history^13^. These models and similar variants have been applied successfully when addressing seasonal to interannual variability at many sites^14–17^, however they may fail to simulate the shoreline evolution in environments where other processes such as alongshore sediment transport play an important role^3^.

Additionally, the equilibrium concept has also been successfully applied to predict shoreline^18^ and sandbar rotation^27^ at pocket beaches. Other longshore transport models^11^ have been applied to long term datasets but lacked the ability to reproduce cross-shore variations^30^. This issue was recently addressed^31–33^, where alongshore sediment transport formulae such as, CERC^34,35^ are combined with cross-shore equilibrium models. Table 1 provides a summary of the HM used during the Shoreshop.

Due to the surge of available measurements characterized by increasing spatial and temporal resolution from camera systems to satellite images^36,37^ and novel approaches to modelling, DDM have become more popular. Examples of these models range from simple autoregressive models to machine learning (ML) techniques such as artificial neural networks. The use of ML techniques in a variety of coastal problems and settings has rapidly increased over the past few years^38^, since ML algorithms can be highly effective predictors^39,40^, can be used as part of larger models^41^ and can provide physical insight^42^. Statistical models such as multiple linear regression or statistical downscaling^43,44^, also fall in the category of data-driven, but have not been tested in the *Shorecast*. One of the drawbacks of DDM is that their performance depends highly on the quantity and quality of the data available. The ML techniques used during the Shoreshop are listed in Table 1. Details on *ML* models are provided in the Supporting Information.

## Supplementary information

Supporting Information

## Data Availability

All data provided to the participants of the “Shoreshop” is available at https://coastalhub.science/data. Other data that support the findings of this study are available from the indicated sources or from the corresponding author upon reasonable request.
