# Supplementary material for: Blind testing of shoreline evolution models
Source: Sci Rep. 2020 Feb 7;10:2137. doi: 10.1038/s41598-020-59018-y (PMC7005834; doi:10.1038/s41598-020-59018-y)
Supplement: Supplementary file 1 — Supporting Information [file 41598_2020_59018_MOESM1_ESM.docx]

**Supporting Information for**

**Blind testing of shoreline evolution models**

Jennifer Montaño^1,*^, Giovanni Coco^1^, Jose A.A. Antolínez^2^, Tomas Beuzen^3^, Karin R. Bryan^4^, Laura Cagigal ^1,2^, Bruno Castelle^5^, Mark A. Davidson^6^, Evan B. Goldstein^7^, Raimundo Ibaceta^3^, Déborah Idier^8^, Bonnie C. Ludka^9^, Sina Masoud-Ansari^1^, Fernando J. Méndez^2^, A. Brad Murray^10^, Nathaniel G. Plant^11^, Katherine M. Ratliff^10^, Arthur Robinet^5,8^, Ana Rueda^2^, Nadia Sénéchal^5^, Joshua A. Simmons^3^, Kristen D. Splinter^3^, Scott Stephens^12^, Ian Townend^13^, Sean Vitousek^14, 15,^ Kilian Vos^3^

^1^ School of Environment, Faculty of Science, University of Auckland, Auckland, 1142, New Zealand

^2^ Departamento de Ciencias y Tecnicas del Agua y del Medio Ambiente, Universidad de Cantabria, Santander, Spain,

^3^ Water Research Laboratory, School of Civil and Environmental Engineering, UNSW Sydney, NSW, 2052, Australia

^4^ School of Science, University of Waikato, Private Bag 3105, Hamilton, New Zealand

^5^ UMR EPOC, University of Bordeaux/CNRS, Bordeaux, France

^6^ Coastal Processes Research Group, School of Biological and Marine Sciences, Plymouth University, Drake Circus, PL4 8AA Plymouth, UK

^7^ Department of Geography, Environment, and Sustainability, University of North Carolina at Greensboro, NC 27412, USA

^8^ BRGM, 3 avenue Claude Guillemin, 45060 Orléans cédex, France

^9^ Scripps Institution of Oceanography, University of California, San Diego, United States

^10^ Division of Earth and Ocean Sciences, Nicholas School of the Environment, Center for Nonlinear and Complex Systems, Duke University Durham, NC, USA

^11^ U.S. Geological Survey St. Petersburg Coastal and Marine Science Center, 600 4th Street South, St. Petersburg, FL, USA

^12^ National Institute of Water and Atmosphere, Hamilton, New Zealand

^13^ University of Southampton, Southampton SO17 1BJ, UK

^14^ Pacific Coastal and Marine Science Center, U.S. Geological Survey Santa Cruz, CA, USA

^15^ Department of Civil and Materials Engineering, University of Illinois at Chicago, IL, USA

Here we provide a brief description of the models used during the *Shoreshop* and a summary of the models' performance assessment based on different metrics.

**Hybrid(HB) Models**

**ShoreFor (HM1)**

ShoreFor is an equilibrium-based cross-shore model first presented in Davidson et al.^1^. The model formulation used in this work follows the modifications of Splinter et al. ^2^ allowing for a more general equilibrium model with inter-site variability of model coefficients. The model formulation follows:

$$\frac{dY}{dt}=c\left( F^{+}+r F^{-} \right)+b$$

Where $\frac{dY}{dt}$ is the rate of shoreline change, dependent on the magnitude of wave forcing $F$ defined as$:$

$F=P^{0.5}\left( \Omega_{\emptyset}-\Omega\right)/\sigma$,

where $P$is the breaking wave energy flux and $\Omega$ is the dimensionless fall velocity. The model includes two coefficients. The first one, c which is the rate parameter accounting for the efficiency of cross-shore sediment transport and $\emptyset$which defines the window width of a filter function, performing a weighted average of the antecedent dimensionless fall velocity and is a proxy for the ‘beach memory’. The model contains two constants, $r={\sum F^{+}}/{\sum F^{-}}$ and $\sigma$ which is the standard deviation of $\left( \Omega_{\emptyset}-\Omega\right)$, both computed over the calibration segment of the wave data. The linear trend parameter, b, has been included to simplistically account for longer-term processes (e.g. longshore sediment transport, sediment supply, etc) not explicitly accounted in the model. The model is calibrated by choosing the minimum normalized mean square error (NMSE) of a least-squares regression solving for c, and b for different values of $\emptyset$in the range of 5 to 1000 days.

**ShoreFor_LX (HM2-R1)**

The ShoreFor_LX model presented here aims to provide a more physical representation of the impact of longshore sediment transport gradients on shoreline change than ShoreFor and is a simple variation of the ShoreFor model described above with the inclusion a CERC-type rotational term in place of the trend term (b):

$$\frac{dY}{dt}=c_{1}\left( F^{+}+r F^{-} \right)+c_{2}\frac{dF_{2}}{dx}$$

Here x is the longshore direction and F2 is the longshore forcing term defined as $P sin\left( 2\alpha\right)$, where $\alpha$ is the angle of wave incidence. This version of the ShoreFor model has an equivalent number of free parameters to the original version (c1, c2 and $\emptyset)$. The model equation is integrated using a standard one-line algorithm^3^.

**Y09-HF(HM3)**

The model is a modification of Yates et al.^4^ to predict faster shoreline changes. The Ensemble Empirical Mode Decomposition (CEEMD) method^5,6^, designed to identify oscillations in non-linear and non-stationary time-series, was applied to the time-series of waves and shoreline position. The Yates model was then applied only to modes with a timescale larger than 60 days, while faster oscillations (between 10-60 days) were considered adding a new term to the Yates model (which involves adding a new free parameter):

$$\frac{dY}{dt}= C^{\pm}E^{0.5}\left( E-E_{eq} \right)+C_{hf}E_{hf}^{0.5}$$

Where the subscript $hf$represents the high frequency oscillations obtained with the CEEMD method. During the calibration, coefficients were selected by minimizing RMSE.

**Shorefor + uKF (HM4)**

This model uses a joint unscented Kalman Filter^7^ within the ShoreFor model (HM1) to assess the time-varying free parameters (*b, c*, $\varphi$) that best fit the observed shorelines during the calibration period. A state vector, here represented by the observed shorelines and the ShoreFor paramaters are sampled through sigma points in a way that their mean and covariance are maintained^8^. The nonlinear system (here given by the original ShoreFor formulation) is used to propagate each sigma point at the prediction step, weighting these propagations to obtain an accurate estimation of the state vector mean and covariance. Then, an update step combines the propagated non-linearities with observed shoreline measurements to optimally estimate the state vector when observed shorelines become available.

Following^9^, we define the total shoreline position $Y(t)$ by the contribution of short-term processes $Y_{st}$ and long-term (or unresolved) processes $Y_{lt}$, thus linking  $Y_{st}$ with the equilibrium formulation and  $Y_{lt}$ with the *b* coefficient.

**Yates et al., 2009 - *3 Coefficients* (HM5)**

HM5 uses the formulation of Yates model, but with a single scalar value for the rate coefficient $C$. (Yates model used a different value of $C$ for erosion and accretion, $C\mp$)

Based on the equilibrium framework suggested by ^10,11^ and similar to Miller and Dean model ^12^, the shoreline change rate depends on both the incident wave energy and the difference between the incident wave energy and the equilibrium energy

$$\frac{dY}{dt}=CE^{0.5}(E-E_{eq})$$

where the equilibrium energy is defined using a linear relationship with the present shoreline position,

$$E_{eq}=aY+b$$

The three free parameters ($a,b$, and $C$) are optimized by minimizing RMSE, using simulated annealing. This model attempts to minimize RMSE while minimizing the number of free parameters, minimizing the number of independent variables (e.g. only wave height is used and not wave period), and minimizing equation complexity (e.g. uses a linear relation for equilibrium energy rather than a quadratic).

**Yates et al, 2009-*4 Coefficients* (HM6, HM7)**

The fitting and hindcasting of shoreline position were done using a derivative of the Kriebel and Dean model^13^, as proposed by Yates et al.^4^. An offset to the initial shoreline was included as an additional fit parameter, as proposed by ^14^. The model relates nearshore wave energy, (represented by $\frac{H_{s}^{2}}{16}$), to shoreline position, $Y$. Inshore wave data were obtained using plane bed refraction to account for refraction and shoaling to the closure depth of the beach profile, taking account of water levels variations due to the tide.

Fitting of the parameters is sensitive to the initial guess and search bounds. Two of the model parameters ($a$ and $b$) are estimated by finding the line for $dY/dt=0$ from the energy between surveys and the shoreline anomaly $(dY)$ and this was modified to use only the points that were close to $dY/dt=0$. The accretion and erosion rates, $C^{+}$ and $C^{-}$, were estimated from the mean positive and negative values of $dY/dt$ respectively, with an initial offset of zero.

Parameter fitting was done using derivative free, constrained non-linear optimisation. Matlab functions for particle swarm, simulated annealing and simplex optimisation were used. Statistically, there was little to choose between them and the results reported used the simplex method. Selection of the best fit was based on minimising the RMSE with the slope of the modelled v observed regression line as close to 1 as possible. In all model runs the large erosional anomalies were better represented than the accretional anomalies, suggesting that the model is not capturing the full extent of beach recovery.

**LX-Shore (HM8, R2)**

LX-Shore is a two-dimensional plan-view cellular-based one-line shoreline change model for wave-dominated sandy coasts presented first in Robinet et al.^15^. The model can simulate shoreline change resulting from gradients in total longshore sediment transport and/or from cross-shore transport driven by the variability in incident wave energy. LX-Shore can handle complex shoreline geometries (e.g. sand spits, islands), including non-erodible areas such as coastal defences and headlands, and is coupled with the spectral wave model SWAN^16^ to cope with complex nearshore wave fields. For the present application, the longshore sediment transport along the coastline was computed using the formula of ^17^ with a calibration multiplication factor (fQl) tuned to minimize the RMSE of beach orientation at Tairua over 1999-2013. The cross-shore transport is resolved using an adaptation of the ShoreFor model^1,2^ where the disequilibrium term is computed from offshore wave conditions instead of breaking wave conditions. The three ShoreFor free parameters $(\varphi$, $c$ and $b$) were optimized using a simulated annealing procedure minimizing the RMSE of alongshore-averaged cross-shore shoreline position at Tairua over 1999-2013. During the simulation, waves were propagated onshore using SWAN with default processes and parameter values. Overall, only 4 calibration parameters were tuned to simulate shoreline change along the entire embayment.

**CosMos-Coast (HM9, R3)**

CoSMoS-COAST is a “one-line” model that integrates longshore and cross-shore transport processes to predict long-term coastal change presented in Vitousek et al.^18^. The model synthesizes several popular process-based shoreline change models including (1) a “one-line” model for longshore transport^19^, (2) a wave-driven cross-shore equilibrium shoreline change model^4^, (3) a cross-shore equilibrium beach profile change model due to sea-level rise^20–22^, and a residual term obtained via data assimilation. The governing equation of CoSMoS-COAST, based on conservation of sediment in the alongshore direction, is given by

$$\underbrace{\frac{\partial Y}{\partial t}}_{\text{shoreline change}}=\underbrace{-\frac{1}{d}\frac{\partial Q}{\partial X}}_{\text{longshore transport}}+\underbrace{CE^{1/2}\Delta E}_{\text{cross-shore transport}}-\underbrace{\frac{c}{\tan\beta}\frac{\partial S}{\partial t}}_{\begin{matrix} \text{shoreline migration} \\ \text{due to sea-level rise} \end{matrix}}+\underbrace{v_{\text{lt}}}_{\begin{matrix} \text{long-term shoreline trend;} \\ \text{unresolved processes} \end{matrix}}$$

The original CoSMoS-COAST model applied an extend Kalman filter data-assimilation technique following^9^. The current version of CoSMoS-COAST applies an ensemble Kalman filter data assimilation technique (with$N=100$ ensembles) following ^23^.

**COCOONED(HM10, R4)**

COCOONED model presented by Antolínez et al.^24^ is a transect-based process-driven coastal change model loaded by waves and varying water levels that solves: nearshore wave propagation with a hybrid scheme based on SWAN^16^; cross-shore transport and equilibrium shoreline change using a modified version of Miller and Dean model^12^; longshore transport and shoreline change with a one line approach similar to^25^; foredune erosion using the model proposed by ^26^ based on ^13^ and the inclusion of profile adjustment by sediment supply. In Tairua, we do not compute dune erosion and we solve the equation:

$$\frac{\partial Y}{\partial t}=\frac{-1}{d}\frac{\partial Q_{L}}{\partial x}+K_{C}\left( Y_{\mathrm{eq}}-Y \right)+\frac{-1}{d}\left( q_{x}+q_{y} \right)$$

where $Y$ represents the shoreline position, $t$ is time, $\frac{q_{x}}{d}$ and $\frac{q_{y}}{d}$ are the alongshore and cross-shore sediment sources per unit shoreline and unit time. $Q_{L}$ is the alongshore transport rate,

$$Q_{L}=Q_{0sin}\left( 2\left( \theta_{b}-\alpha_{\mathrm{shoreline}} \right) \right)$$

where $Q_{0}$ is computed applying the CERC^27^ sediment transport formula. The cross-shore component of the model is based upon the general observation that the shoreline tends to approach an equilibrium position, $Y_{\mathrm{eq}}$.

The model is set-up with transects every 100 m and is run with a time step of 6h. There are no sediment sources at the boundaries ($q_{x}=0$, $q_{y}=0$), and we use the waves characteristics are provided. The model is loaded with waves and water levels. Three calibration parameters are set up to reproduce the observational period provided in terms of RMSE, bias and correlation coefficient (alongshore transport rate, $K_{L}$; erosional cross-shore transport rate, $K_{C,e}$; accretional cross-shore transport rate, $K_{C,a}$) at every transect.

**Rotation R5, R6**

These models use the alongshore wave energy flux to predict the orientation of the shoreline, where the orientation is the slope of the regression line fit to the shoreline for each time step. The models are essentially the same as the one-line diffusion models (reviewed in ^28^), which have been applied to beach rotation in ^29^,with the addition that the beach rotates around its alongshore equilibrium position (which is the shoreline that would evolve if the alongshore wave energy flux was zero.) As the alongshore wave energy flux deviates from zero, there is a time-delay over which the beach responds to the change. ^29^ use the beach volume and sediment characteristics to inform estimates of the delay. We use the version in ^30^ where the rotation rates are coefficients $C$ that are set by fitting the observations to the model.

$$\frac{\partial\alpha}{\partial t}=CE\left( \sin\theta\cos\theta-\alpha cos 2\theta\right)$$

Where E is wave energy, θ is wave approach angle and α is the shoreline orientation. In R5, constant coefficients were used, with a different one for negative and positive wave energy flux. In R6, coefficients were linearly dependant on the wave energy flux, with the slope and intercept fitted by matching model output to observations (this should allow better fitting of large events). The optimal values of coefficients were obtained by RSME using boot-strapping for optimisation. The mean was removed from the observations and mean beach orientation (54.2 degrees) was removed from θ.

**Machine Learning (ML) Models**

**k Nearest Neighbor (kNN)**

The k-nearest neighbor (kNN) model used the past as a ‘catalog’ to predict the future system dynamics. Here, the ‘catalog’ is a set of 5 dimensional vectors, where each vector represents a day. The 5 dimensions are the daily significant wave height, Hs, daily shoreline position, two past shoreline positions (from previous days) and the future shoreline positions (which can be used to compute daily shoreline change). A prediction involves searching the catalog to find the ‘k’ nearest vectors in the 4 dimensional Euclidean space. The ‘k’ past instances are used to compute a weighted average (using inverse distance) of the daily shoreline change. The shoreline position can then be updated, and the whole process repeats for the following day’s prediction. To optimize parameters, the known historic data were split into two parts — the first was used as a catalog, and the second was treated as validation data to find optimized parameter values. After the optimization, the entire catalog of known data were used for the ‘Shorecast’.

**Autoregressive neural network (ANN) with exogenous inputs**

The input dataset consisted of a multidimensional dataset combining the modes obtained through out the Complete Ensemble Empirical Mode Decomposition method ^5^, with daily wave conditions ($H_{s},$mean wave period$T_{02}$, wave direction$\theta$) and the shoreline position of the previous day$Y_{i-1}$. The two algorithms proposed used a different number of nodes (7 and 2) to connect the variables in a single hidden layer (a sigmoid function was used to connect different layers). The calibration was performed by minimizing the RMSE and differed between the two algorithms proposed. In the second algorithm, white noise (+/- 5 m) was added to the shoreline signal to minimize overfitting.

**Long-Short Term Memory (LSTM)**

LSTMs are recurrent neural network models capable of learning long-term dependencies in sequence modelling problems ^31^. A stateless model was created using six input features $(H_{s},T_{01},T_{02},T_{p}, \theta$and the previous shoreline position $Y_{i-1}).$Features were normalised and training samples were generated adding sequence subsampling with a window size of 10 time-steps The LSTM model was implemented using Python with Keras and Tensorflow. The model architecture consisted of a single LSTM unit fully connected to a single output neuron using a linear activation. Mean Squared Error was used as the loss function and the Adam^32^ optimizer was used to tune network weights. Nested cross-validation was performed using 5 partitions with a batch size of 4 and 10 epochs per training cycle. In all cases the random number generator was initialised to 42. Daily forecasts were generated iteratively, using the forecasted value as part of the next input series.

**NeuFor**

A neural network (multilayer perceptron) was used to predict shoreline change ($dY_{t}$) at each timestep ($t$) and integrated to model shoreline position ($Y_{t}$) over time. The neural network takes as input the normalised hydrodynamic forcing ($H_{s}$ and $T_{02}$) over a daily shoreline change timestep. The model has an explicit feedback whereby the previous prediction of overall shoreline position ($Y_{t-1}$) forms an additional normalised input variable to the network. The hydrodynamic forcing variables are provided to the model at three-hour timesteps making a total of 17 input variables including the previous shoreline position. An exhaustive search using 5-fold cross-validation was to determine the optimum model inputs, architecture and hyperparameters. The model was developed and trained using the Python package PyTorch to optimise mean squared error and the coefficient of determination when predicting shoreline position over time. The final model contained two hidden layers (of 40 and 20 neurons), with rectified linear unit activation functions and a dropout of 40% on each of the hidden layers to prevent overfitting.

**Random Forest (RF)**

Random Forests (RFs) are ensembles of decision trees^33^. Training time series of shoreline positions were linearly interpolated and smoothed with a three day moving average. The input variables used are $H_{s}$, $T_{p}$and the disequilibrium in dimensionless fall velocity ${(\Omega}_{dis})$ defined as the difference between the instantaneous and weighted average of the antecedent dimensionless fall velocity^1^ and using a ‘memory decay’ (ϕ) of 30 days. $H_{s}$, $T_{p}$ and $\Omega$ were defined at 12 and 24 hours prior to time *t*, resulting in a total of seven input variables. We used the Python toolkit SciKit-Learn^34^. The final model was an ensemble of 50 decision trees where each tree was constructed using a bootstrap sample of the training dataset. The samples were the same size as the training data but drawn with replacement. Each tree was also constrained to have a maximum depth of eight and a minimum of 20 data samples was required to form a split in the tree. The hyperparameter values were selected following an exhaustive 10-fold cross-validation grid search with the RF performance measured as the RMSE from 10-fold cross-validation.

**Bayesian Networks (BN)**

Following ^35^ we forecast the alongshore averaged shoreline position using as input variables the position at an earlier time $(Y_{i-1})$, $H_{s}$, $T_{02}$, and wave direction. Model time-step was 5 days, and faster variations were removed by time-averaging over this interval. Time-averaged inputs were used to train the BN^36^. Input variables were discretized into 3 bins, each having equal prior probability based on the training data. The output bin ($Y_{i+1})$, was discretised into 5 bins. A 10-fold calibration/validation testing approach ^37^ ensured that forecast skill was maximized. During calibration, the RMSE was minimized but results indicate that the calibration included some overfitting. We cast the forecast into a Bayesian-mean value, $x$, where $x=\sum p\left( i \right)x(i)$ over the bins, $p\left( i \right)$ is the predicted forecast probability that the shoreline lies in the *ith* bin and $x\left( i \right)$ is the shoreline position at the center of the *ith* bin. The forecast uncertainty can be computed as the variance using the same approach. The results obtained at the model time step (5 days) were linearly interpolated to the sample time step (1 day) for comparison with the other models.

**Models performance assessment.**

A brief description of the metrics used is provided below. The following symbols are used:

- $o=$ observed shoreline position /rotation angle

- $m=$modelled shoreline position /rotation angle.

Also,the use of a top bar ($\bar{x)}$ indicate the average, $\sigma_{x}$ denotes the standard deviation,$\sigma_{x}^{2}$ is the variance, $\left| x \right|$ is the absolute value and $N$ the length of the time series. We used the following metrics:

- *Squared error or coefficient of determination:* Indicates how well model fit the observed data in terms of their correlation. A disadvantage $R^{2}$ is that the series may be well correlated but have large residuals^1^.

${R^{2}=\left( \frac{\mathbf{1}}{\boldsymbol{N}-\mathbf{1}}\sum_{\boldsymbol{i}=\mathbf{1}}^{\boldsymbol{N}} \frac{\left( \boldsymbol{o}_{\boldsymbol{i}}-\bar{\boldsymbol{o}} \right)\left( \boldsymbol{m}_{\boldsymbol{i}}-\bar{\boldsymbol{m}} \right)}{\boldsymbol{\sigma}_{\boldsymbol{o}}\boldsymbol{\sigma}_{\boldsymbol{m}}} \right)}^{\mathbf{2}}$.

- *Root Mean Square Error:* Summarize the mean differences in units of o and m.

$RMSE= \sqrt{\frac{1}{N}\sum_{i=1}^{N} \left( o_{i}-m_{i} \right)^{2}}$

- *Skill:* Compares the error variance with the observed variance by normalizing RMSE. In general, skill can range as: a poor model performance (0-0.2), Fair(0.2-0.4) , Good(0.4-0.7), Excellent(> 0.7).

$$Skill=1-\frac{\sum_{i=1}^{N} \left( o_{i}-m_{i} \right)^{2}}{\sigma_{o}^{2}}$$

- *Mean Absolute Error:* As the RMSE, this metric does not consider the direction of the errors and additionally it is unbounded.

$MAE= \frac{1}{N}\sum_{i=1}^{N} \left| o_{i}-m_{i} \right|$.

*Index of Agreement d*^38^*:* The index of agreement can detect additive and proportional differences in the observed and simulated means and variances. However, it is overly sensitive to extreme values due to the squared differences.

$$d=1- \frac{\sum_{i=1}^{N} \left( o_{i}-m_{i} \right)^{2}}{\sum_{i=1}^{N} \left( \left| m_{i-}\bar{o} \right|+\left| o_{i-}\bar{o} \right| \right)^{2}}$$

where $0\leq d\leq1$ , with values close to zero for poor performance and values close to 1 indicating excellent performance.

- *Mielke’s modification* $\boldsymbol{\lambda}$ ^39^*:* It is a symmetric index, dimensionless and bounded as the index of agreement.

$$\lambda=1- \frac{N^{-1}\sum_{i=1}^{N} \left( o_{i}-m_{i} \right)^{2}}{\sigma_{o}^{2}+\sigma_{m}^{2}+{(\bar{o}-\bar{m})}^{2}}$$

Table S3 displays the above metrics for each model. Each model is represented by a colour shown in the legend. Models that obtained a good performance based on each metric are in the upper part of the table.

**Table 1.** Summary of different metrics used to assess models performance. Upper panels display values for the averaged alongshore shoreline position and bottom panels metrics for the rotation predictions during the calibration period (left) and Shorecast (right).

**References**

1. Davidson, M. A., Splinter, K. D. & Turner, I. L. A simple equilibrium model for predicting shoreline change. *Coast. Eng.* **73,** 191–202 (2013).

2. Splinter, K. D. *et al.* A generalized equilibrium model for predicting daily to interannual shoreline response. *J. Geophys. Res. F Earth Surf.* **119,** 1–23 (2014).

3. Kamphuis, J. W. *Introduction to coastal engineering and management*. (World Scientific, 2010).

4. Yates, M. L., Guza, R. T. & O’Reilly, W. C. Equilibrium shoreline response: Observations and modeling. *J. Geophys. Res. Ocean.* **114,** 1–16 (2009).

5. Huang, N. E. *et al.* The empirical mode decomposition and the Hilbert spectrum for nonlinear and non-stationary time series analysis. *Proc. R. Soc. A Math. Phys. Eng. Sci.* **454,** 903–995 (1998).

6. Torres, M., Colominas, M. A., Schlotthauer, G. & Flandrin, P. A complete ensemble empirical mode decomposition with adaptive noise. in *2011 IEEE international conference on acoustics, speech and signal processing (ICASSP)* 4144–4147 (2011).

7. Wan, E. A. & van der Merwe, R. The Unscented Kalman Filter. in *Kalman Filtering and Neural Networks* 221–280 (2001).

8. Julier, S. J. & Uhlmann, J. K. Unscented filtering and nonlinear estimation. in *Proceedings of the IEEE 92.3* 401–422 (2004).

9. Long, J. W. & Plant, N. G. Extended Kalman Filter framework for forecasting shoreline evolution. *Geophys. Res. Lett.* **39,** 1–6 (2012).

10. Wright, L. D. & Short, A. D. Morphodynamic variability of surf zones and beaches: a synthesis. *Mar. Geol.* **56,** 93–118 (1984).

11. Wright, L. D., Short, A. D. & Green, M. O. Short-term changes in the morphodynamic states of beaches and surf zones: An empirical predictive model. *Mar. Geol.* **62,** 339–364 (1985).

12. Miller, J. K. & Dean, R. G. A simple new shoreline change model. *Coast. Eng.* **51,** 531–556 (2004).

13. Kriebel, B. D. L. & Dean, R. G. Convolution method for time-dependent beach-profile response. *J. Waterw. Port, Coastal, Ocean Eng.* **119,** 204–226 (1993).

14. Castelle, B. *et al.* Equilibrium shoreline modelling of a high-energy meso-macrotidal multiple-barred beach. *Mar. Geol.* **347,** 85–94 (2014).

15. Robinet, A., Idier, D., Castelle, B. & Marieu, V. A reduced-complexity shoreline change model combining longshore and cross-shore processes: The LX-Shore model. *Environ. Model. Softw.* **109,** 1–16 (2018).

16. Booij, N. R. R. C., Ris, R. C. & Holthuijsen, L. H. A third‐generation wave model for coastal regions: 1. Model description and validation. *J. Geophys. Res. Ocean.* **104,** 7649–7666 (1999).

17. Kamphuis, J. W. Alongshore Sediment Transport Rate. *J. Waterw. Port, Coastal, Ocean Eng.* **117,** 624–640 (1991).

18. Vitousek, S., Barnard, P. L., Limber, P., Erikson, L. & Cole, B. A model integrating longshore and cross-shore processes for predicting long-term shoreline response to climate change. *J. Geophys. Res. Earth Surf.* 1–25 (2017). doi:10.1002/2016JF004065

19. Pelnard-Considère, R. Essai de théorie de l’évolution des formes de rivage en plages de sable et de galets. *Les Energies de la Mer: Compte Rendu Des Quatriemes Journees de L’hydraulique* 13 (1956).

20. Bruun. Sea-Level Rise as a Cause of Shore Erosion. *J. Waterw. Harb. Div.* **88,** 117–132 (1962).

21. Davidson-Arnott, R. G. D. Conceptual Model of the Effects of Sea Level Rise on Sandy Coasts. *J. Coast. Res.* **216,** 1166–1172 (2005).

22. Anderson, T. R., Fletcher, C. H. ., Barbee, M. M., Frazer, L. N. & Romine, B. M. Doubling of coastal erosion under rising sea level by mid-century in Hawaii. *Nat. Hazards* **78,** 75–103 (2015).

23. Evensen, G. Sequential data assimilation with a nonlinear quasi-geostrophic model using Monte Carlo methods to forecast error statistics. *J. Geophys. Res. Ocean.* **99,** 10143–10162 (1994).

24. Antolínez, J. A. A., Méndez, F. J., Anderson, D., Ruggiero, P. & Kaminsky, G. M. Predicting climate driven coastlines with a simple and efficient multi‐scale model. *J. Geophys. Res. Earth Surf.* 2018JF004790 (2019). doi:10.1029/2018JF004790

25. Vitousek, S. & Barnard, P. L. A nonlinear, implicit one-line model to predict long-term shoreline change. in *Proceedings of the Coastal Sediments 2015* (2015).

26. Mull, J. & Ruggiero, P. Estimating Storm-Induced Dune Erosion and Overtopping along U.S. West Coast Beaches. *J. Coast. Res.* **298,** 1173–1187 (2014).

27. USAGE. *Shore protection manual*. (1984). doi:doi.org/ 10.5962/bhl.title.47829

28. Ruggiero, P., Buijsman, M., Kaminsky, G. M. & Gelfenbaum, G. Modeling the effects of wave climate and sediment supply variability on large-scale shoreline change. *Mar. Geol.* **273,** 127–140 (2010).

29. Turki, I., Medina, R., Coco, G. & Gonzalez, M. An equilibrium model to predict shoreline rotation of pocket beaches. *Mar. Geol.* **346,** 220–232 (2013).

30. Blossier, B., Bryan, K. R., Daly, C. J. & Winter, C. Shore and bar cross-shore migration, rotation, and breathing processes at an embayed beach. *J. Geophys. Res. Earth Surf.* **122,** 1745–1770 (2017).

31. Hochreiter, S. & Schmidhuber, J. Long short-term memory. *Neural Comput.* **9,** 1735–1780 (1997).

32. Kingma, D. P. & Ba, J. Adam: A Method for Stochastic Optimization. in *arXiv preprint arXiv:1412.6980* 1–15 (2014).

33. Breiman, L. Random forests. *Mach. Learn.* **45,** 5–32 (2001).

34. Pedregosa, F. *et al.* Scikit-learn: Machine learning in Python. *J. Mach. Learn. Res.* **12,** 2825–2830 (2011).

35. Plant, N. G. & Stockdon, H. F. Probabilistic prediction of barrier-island response to hurricanes. *J. Geophys. Res. Earth Surf.* **117,** 1–17 (2012).

36. Corp, N. S. Netica 5.05.

37. Fienen, M. N. & Plant, N. G. A cross-validation package driving Netica with python. *Environ. Model. Softw.* **63,** 14–23 (2015).

38. Willmott, C. J. & Willmott, C. J. Some Comments on the Evaluation of Model Performance. *Bull. Am. Meteorol. Soc.* **63,** 1309–1313 (1982).

39. Duveiller, G., Fasbender, D. & Meroni, M. Revisiting the concept of a symmetric index of agreement for continuous datasets. *Sci. Rep.* **6,** 1–14 (2016).
